# Adult Development and Aging in Historical Context

**DOI:** 10.1093/geroni/igaa057.2027

**Published:** 2020-12-16

**Authors:** Denis Gerstorf, Johanna Drewelies, Sandra Duezel, Hans-Werner Wahl, Corinna Löckenhoff, Ilja Demuth, Nilam Ram

**Affiliations:** 1 Humboldt University Berlin, Berlin, Berlin, Germany; 2 Humboldt University Berlin, Berlin, Germany; 3 Max Planck Institute for Human Development, Berlin, Berlin, Germany; 4 University of Heidelberg, Heidelberg, Baden-Wurttemberg, Germany; 5 Cornell University, Ithaca, New York, United States; 6 Charité – Universitätsmedizin Berlin, Berlin, Berlin, Germany; 7 Pennsylvania State University, University Park, Pennsylvania, United States

## Abstract

Human development is shaped by socio-cultural contexts and the historical changes therein. Empirical reports suggest that old age has gotten “younger”, both on behavioral measures and in people’s perception. Here, we move one step further and shed light on key quality of life facets not yet well understood. We compare matched cohorts (each n = 250, Mage = 77) assessed 25 years apart in the Berlin Aging Studies (1990–93 vs. 2017–18). Extending the evidence to personality, older adults today are on average lower on neuroticism and higher on openness than their age peers in the past. Qualifying the picture, no evidence emerged for historical changes across four indices of perceptions of aging. Rounding out the picture, we also observed that older adults today perceive more time pressure than age peers 25 years ago. We discuss implications of this nuanced picture of historical changes among older adults.

